# Supplementary material for: The Construction and Exploration of a Comprehensive MicroRNA Centered Regulatory Network in Foxtail Millet (Setaria italica L.)
Source: Front Plant Sci. 2022 May 6;13:848474. doi: 10.3389/fpls.2022.848474 (PMC9121102; doi:10.3389/fpls.2022.848474)

T=Seita.9G035800.1_Q=Sit−miRN3027_S=1829

category=2_p=0.749880059799668

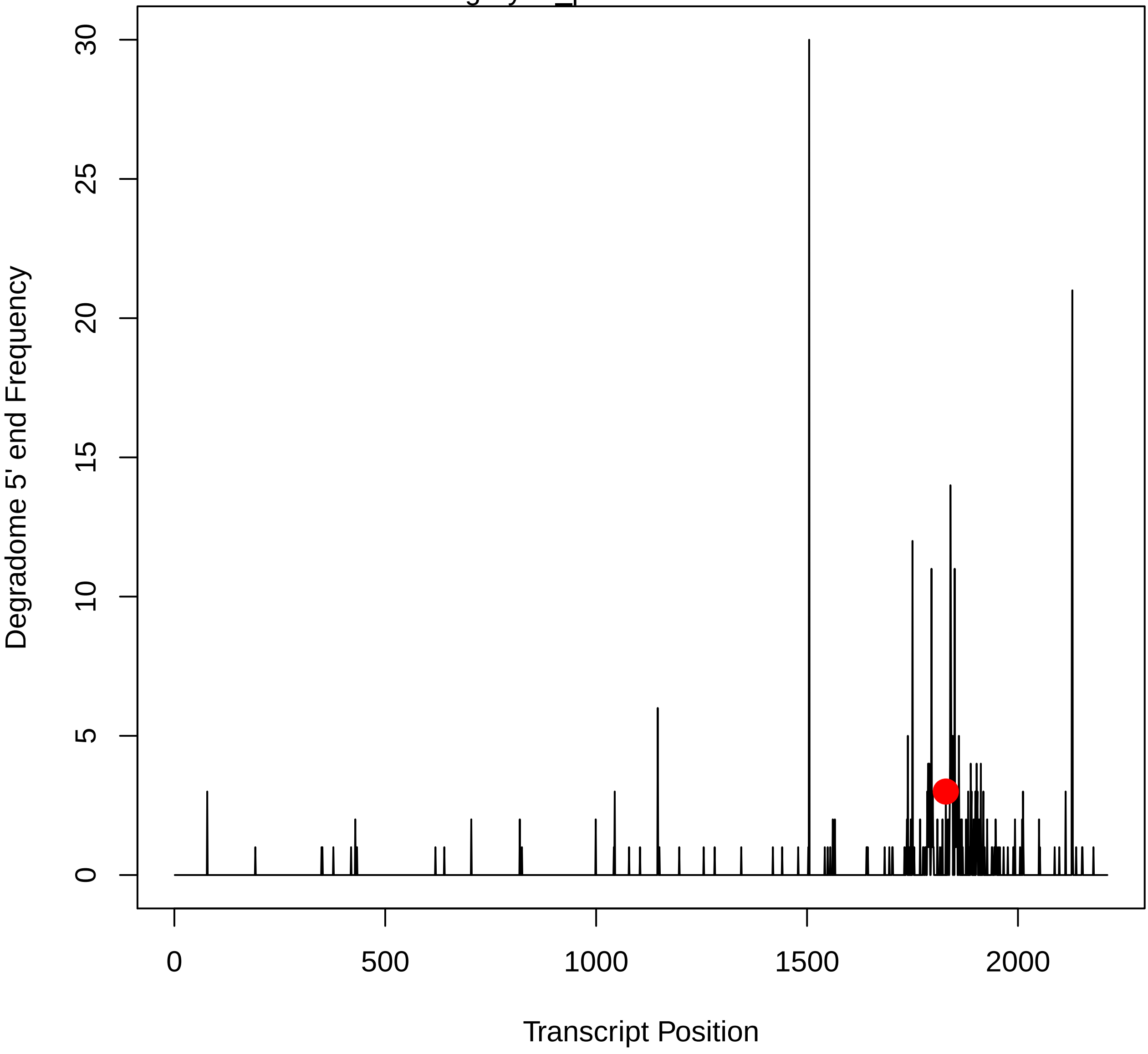

Supplement: Supplementary file 2 [file Data_Sheet_2.zip › Sit-miRN3027_Seita.9G035800.1_1829_TPlot.pdf]
